# Risk Assessment of Micronutrients Deficiency in Vegetarian or Vegan Children: Not So Obvious

**DOI:** 10.3390/nu15092129

**Published:** 2023-04-28

**Authors:** Jean-Pierre Chouraqui

**Affiliations:** Paediatric Nutrition and Gastroenterology, Paediatrics Department, University Hospital of Grenoble-Alpes (CHUGA), Quai Yermoloff, 38700 La Tronche, France; chouraquijp@wanadoo.fr

**Keywords:** infants, children, vegetarian, vegan, macrobiotic

## Abstract

Vegetarian diets have gained in popularity worldwide and therefore an increasing number of children may be exposed to the resulting nutritional consequences. Among them, the risk of micronutrient shortfall is particularly of concern. This narrative review aims to assess and discuss the relevance of micronutrient deficiency risk based on the available data. It mainly draws attention to iron, zinc, iodine, and vitamins B12 and D intake. Diets that are more restrictive in animal source foods, such as vegan diets, have a greater likelihood of nutritional deficiencies. However, the actual risk of micronutrient deficiency in vegetarian children is relatively difficult to assert based on the limitations of evidence due to the lack of well-designed studies. The risk of vitamin B12 deficiency must be considered in newborns from vegan or macrobiotic mothers and children with the most restrictive diet, as well as the risk of iron, zinc, and iodine deficiency, possibly by performing the appropriate tests. A lacto-ovo-vegetarian diet exposes a low risk if it uses a very varied diet with a sufficient intake of dairy products. Vegan and macrobiotic diets should be avoided during pregnancy and childhood. There is a need for education and nutrition guidance and the need for supplementation should be assessed individually.

## 1. Introduction

Micronutrients are vitamins and minerals that are needed in very small amounts for health (mg or μg/day), and that enable the body to produce enzymes, hormones, and other substances which are essential for proper growth and development [1]. Micronutrients are therefore even more crucial during the phases of rapid growth and development in childhood, i.e., the 1000 first days and first years of life and adolescence [2,3,4,5]. Micronutrient deficiencies may lead to deleterious health conditions, and less clinically noticeable disorders such as lower academic performance and an increased risk of diseases [2,3,6,7]. On the other hand, there is increasing evidence that diets during childhood and adolescence can impact health in later adulthood [8]. The World Health Organization’s (WHO) Ambition and Action in Nutrition 2016–2025 aimed to provide a fit-for-purpose nutrition strategy and recalled the resolution WHA 37.18 on prevention and management of micronutrient malnutrition [9].

Micronutrients can only be provided in the diet and their deficiencies in children without underlying disease are food-borne. In general, micronutrients content tends to be denser, more varied, and more bioavailable in animal-source foods (ASF, including meat, fish, eggs, dairy products, and animal-derived ingredients) than in foods of plant origin (FPO) [6,10,11]. ASF are the almost exclusive dietary sources of vitamin B12 and vitamin D, apart from the possible contributions by certain mushrooms and yeasts. ASF is also the source of highly bioavailable vitamin A (retinol), iron, and zinc. Moreover, they provide a daily intake of riboflavin, choline, and vitamin E. Otherwise, vegetable oil and nuts may represent the most prominent sources of vitamin E. Dietary iodine intake depends mainly on that of iodized salt or otherwise from ASF and to a lesser extent from FPO depending then on the iodine content of the local soil and water.

Populations consuming little or no ASF, i.e., a vegetarian diet, are therefore exposed to the risk of deficiency in some or all these nutrients depending on their level of ASF intake [12]. The degree of ASF restriction as well as that of the permitted foods define the different vegetarian patterns [13,14,15]. All patterns include prolonged breastfeeding as much as possible. The lacto-ovo-vegetarian diet (LOV), very often assimilated to a vegetarian diet, is most often practiced, and excludes meat (all types and derived processed products), fish, and seafood but includes dairy products, eggs, and honey, together with a wide variety of plant foods. In addition, lacto-vegetarians also exclude eggs, while pollotarians consume poultry, pescatarians consume fish and seafood, and ovo-vegetarians may consume eggs but not milk. Occasionally and variably flexitarians will eat meat or fish. Veganism is much more restrictive and excludes all ASF, as well as products containing ingredients derived from ASF and all items of animal origin (e.g., wool, silk, leather). A macrobiotic diet is based on cereals, pulses, vegetables, seaweed, and soy products and may include fish.

As a benchmark, it should be acknowledged that in some parts of the world, mainly in Asia, human beings have been able for millennia not only to survive but also to maintain long and healthy lives on diets free from flesh [16,17,18]. Such diet remains part of the cultural and religious tradition in these countries essentially out of respect for all living beings [19]. Furthermore, avoidance of ASF is increasingly popular in industrialized countries due to a growing concern for animal welfare, sustainable development, and health, not to mention the socioeconomic impact of animal product consumption [15,20,21,22,23,24,25,26,27,28]. In households, children’s eating behavior depends on parents purchasing family food, setting an example, and wanting their children to share their beliefs and eating habits [27,29,30]. On the other hand, more and more establishments such as kindergartens, schools, colleges, restaurants, and hospitals are providing vegetarian options at least one or two days a week for educational and environmental reasons. As a result, the question of the risks and benefits of vegetarian diets in childhood arises. Among the risks, those linked to an insufficient intake of micronutrients constitute a particularly important issue. This narrative review aims to assess and discuss the relevance of micronutrient deficiency risk based on the available data.

## 2. Search Strategy

A comprehensive search of the literature using PubMed, EMBASE, Google Scholar, and the Cochrane Library was conducted from 1980 up to January 2023. The keywords (words or MeSH terms) used were: ‘micronutrients’, ‘vitamin’, ‘mineral’, ‘vegetarian’, or ‘vegan’, ‘pregnancy’, ‘lactation’, ‘breastfeeding’, ‘newborn’, ‘infant’, ‘toddler’, ‘child’, ‘adolescent’, ‘dietary intake’, or ‘deficiencies’. Moreover, a search was undertaken to identify relevant papers referred to in previously identified articles. Original papers, reviews, meta-analyses, position papers, and guidelines published by expert scientific groups or societies were included. Articles in English or with relevant English abstracts and in French were selected. When relevant data were identified in another language, an online translator was used so as not to overlook that information. After reading the title and abstract of the identified articles, duplicate references were removed. Thus, of the 332 publications first selected, 122 are finally cited.

## 3. Vitamins

### 3.1. Vitamin Supply from Foods from Plant Origin

FPO may be a significant source of antioxidant vitamins such as vitamin C (ascorbic acid), E (tocopherol), and provitamin A carotenoids, as well as vitamin K (phylloquinone), but also vitamin B1(thiamine), B2 (riboflavin), B3 (niacin, PP), B5 (pantothenic acid), B6 (pyridoxine), B7 (biotin), B9 (folates) [31,32,33,34]. However, the impact of cooking (heating and leaching) and processing on the available vitamin content should not be neglected [34]. Compared to intakes of these vitamins in omnivorous children, those in vegetarians and vegans have been shown either identical or even higher in most studies, especially in vegans [35,36,37,38,39,40,41,42,43,44,45,46,47,48,49]. Lower intake of vitamin B2 has been reported in children following a macrobiotic diet compared to omnivores [35]. No studies assessed the vitamin K or vitamin B5 intake.

On the other hand, the dietary intake of vitamin D is very limited and can only come from infant’s and young child’s formulas and to a lesser extent from some fatty fishes or even less from some mushrooms [50,51,52,53,54]. Compared to the intake in omnivorous children, no study found a difference in vegetarian children [45,46,47], but one found a lower intake in vegan children [41].

The major issue in vegetarian diets is related to the total lack of vitamin B12 in FPO [55]. The only possible plant source of vitamin B12 could be some algae but with a low bioavailability [56,57]

### 3.2. Vitamin B12 Concern

Vitamin B12 is essential for human metabolism acting as a coenzyme in the conversion of methylmalonyl-CoA to succinyl-CoA in propionate metabolism and the transmethylation of homocysteine to methionine [58]. After reviewing published dietary reference values, the European Food Safety Authority (EFSA) sets an adequate dietary intake of cobalamin of 4.5 μg/d for pregnant women, 5 μg/d for lactating women, 0.5 μg/d for infants under 7 months of age, 1.5 μg/d for infants 7–11 months and children aged 1–6 years, 2.5 μg/d for children 7–10 years old, 3.5 μg/d for teenagers 11–14 years old and 4 μg/d for adolescent 15–17 years old. Symptoms and the long-term prognosis of cobalamin deficiency in children depend on the severity and duration of the deficiency [56]. The diagnosis may be difficult in the mild case and in the absence of specific signs which may lead to a substantial diagnosis delay that can be pejorative in terms of prognosis. Practitioners should be aware of this possibility in infants from vegetarian mothers and children on a vegetarian diet who do not take supplements.

#### 3.2.1. Newborns and Infants

The need for vitamin B12 is increased during pregnancy and lactation in relation to the expansion of tissues and supply to the fetus and the newborn [58,59]. When adhering to vegetarian diets for at least 3 years without supplementation, 22% of pregnant women have been shown to be vitamin B12 deficient [60]. Maternal vitamin B12 deficiency is associated with low birth weight in addition to an increased risk of pregnancy complications [61]. Cobalamin status in the first six months of life depends on maternal cobalamin status during pregnancy [55,62]. The offspring of vitamin B12-deficient mothers are usually asymptomatic at birth but may develop clinical signs at the age of 4–6 months [63]. Symptoms may include megaloblastic anemia, feeding difficulties, failure to thrive, irritability, muscular hypotonia, tremors and seizures, and impaired neurodevelopment which may be irreversible [61,63].

The cobalamin concentration of breast milk reflects maternal cobalamin concentration in blood [56]. The main nutritional deficit of breast milk from vegetarian and vegan mothers is that of vitamin B12 [64,65]. A vegetarian diet in the mother is the main cause (64% of cases) of vitamin B12 deficiency in breastfed infants [66]. In India, the country with the highest prevalence of vegetarians [67], 63.7% of 149 exclusively breastfed infants (3.1 ± 1 mo) had low levels of serum vitamin B12 (<200 pg/mL), but only 21% of them had a vegetarian mother [68]. Symptoms of cobalamin deficiency in breastfed infants are usually manifested between 4 to 8 months of age [56,69,70]. Numerous breastfed infants of vegetarian or vegan mothers have been reported to develop severe vitamin B12 deficiency with anemia, failure to thrive, hypotonia, developmental delay, microcephaly, and cerebral atrophy [69,70,71,72,73].

#### 3.2.2. Children and Adolescents

**Figure 1:**
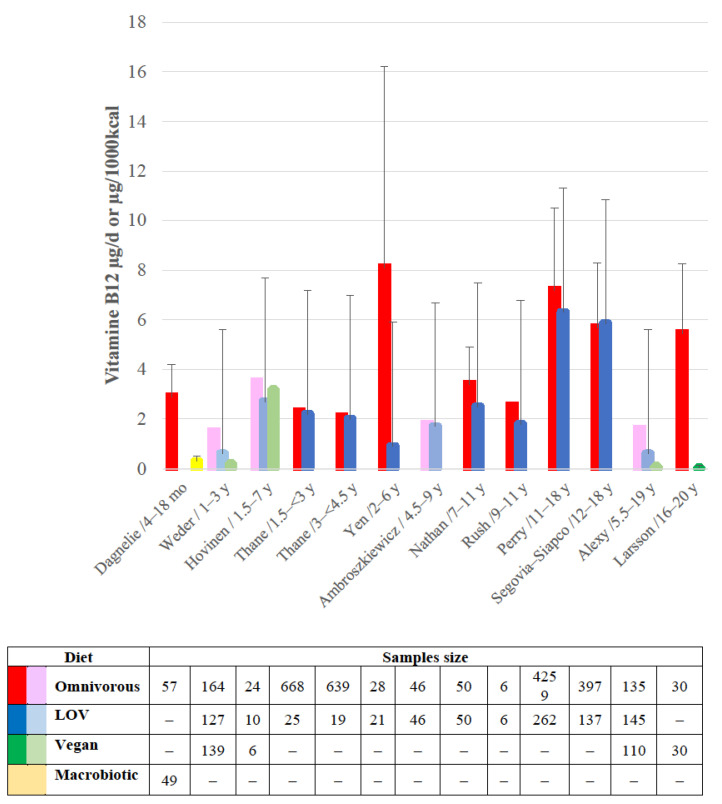
Bar chart to present the reported vitamin B12 intake in lacto-ovo-vegetarian (LOV), vegan or macrobiotic children compared to omnivorous counterparts [35,36,39,40,41,43,46,47,48,49,74,75]. The dark-colored bars display the mean with SD, when available, whereas the light-colored bars display the median value.

The reported intakes of vitamin B12 in children are depicted in Figure 1. The more the diet restricts ASF intake and the longer the compliance with this restriction, the lower the intake [35,36,39,40,41,43,46,47,48,49,74,75].

Many studies have confirmed the existence of a vitamin status deficiency in children by showing a low level of serum cobalamin, and/or an increase in plasma methylmalonic acid or homocysteine [35,74,75,76,77,78]. However, others failed to get the same results, probably due to not taking into account possible supplements, or a short duration of the restrictive diet [39,43,63,74,78,79]. Vitamin B12 deficiency develops slowly given that vitamin B12 may be stored in the liver [76]. Overall, the reported prevalence of vitamin B12 deficiency was 62% in pregnant vegetarians, 25–85% in vegetarian children, and 21–41% in vegetarian adolescents [80]. The greater prevalence of deficiency was in vegans and those who had adhered to a vegetarian diet since birth. Nearly 40% of 210 apparently healthy Indian children aged 6–23 months have been shown to be deficient in vitamin B12 (<210 pg/mL), especially since they had not consumed cow’s milk for at least 6 months (OR 2.6, 95% CI 1.4–4.6) [81]. Severe vitamin B12 deficiency in children on a vegan diet and requiring hospitalization was reported in Italy [82]. In a macrobiotic community, 55% of children were vitamin B12-deficient according to their urinary methylmalonic acid [83]. Overall, deficiency symptoms are the same as those described above in infants [69,84]. In the Indian study enrolling 27 young children (6–27 months), anemia was found in 83%, developmental delay or regression in all, and cerebral atrophy was found in the 9 children who underwent neuroimaging [84]. Usually, an appropriate diagnosis of vitamin B12 deficiency is based on the coexistence of megaloblastic anemia with neurological disorders. However, given that folate intake is most of the time high in vegetarians, the pathognomonic haematologic characteristics of vitamin B12 deficiency may be masked [76,85].

## 4. Minerals

FPO are relatively rich in certain minerals such as potassium and magnesium but have variable content in zinc, copper, and selenium and only contain non-haeme iron with poor bioavailability [10,31,32,86]. Absorption of iron, zinc, and calcium is reduced by phytates and/or oxalates which are quite abundant in unrefined cereals, whole grains, and legumes [76,86,87,88,89]. However, due to its abundance in the human body, calcium is not considered a micronutrient [90,91,92,93,94]. Inadequate intake and/or poor bioavailability of these microminerals are the main cause of deficiency [90].

### 4.1. Iron

**Figure 2:**
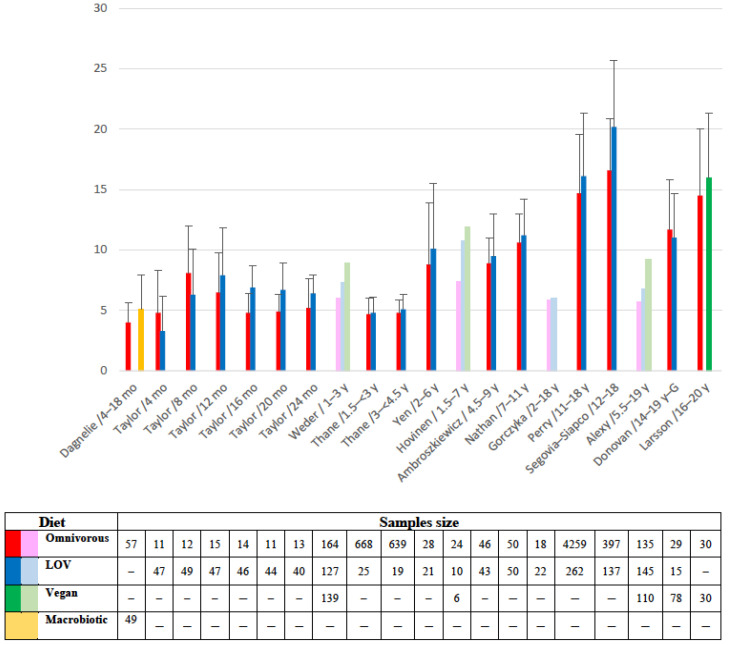
Bar chart presenting the reported iron intake in lacto-ovo-vegetarian (LOV) and vegan children compared to omnivorous counterparts [35,36,37,39,40,41,42,44,46,47,48,49,74,95]. The dark-colored bars display the mean with SD, when available, whereas the light-colored bars display the median value.

The main role of iron in the body is to ensure the oxygen-carrying capacity of hemoglobin and tissue oxygenation. Current knowledge on iron homeostasis, dietary intake, and prevention of iron deficiency has been recently addressed in this journal [86]. Inadequate intake of bioavailable iron may lead to depletion in body stores and iron deficiency which can be assessed by the measurement of serum ferritin in the absence of inflammation. Children are at particular risk of iron deficiency due to their rapid growth. Iron deficiency will precede the onset of iron deficiency anemia which can lead to poor neurodevelopment. Dietary iron intake and absorption are the main factors driving body iron status alongside blood losses. Most FPO have low iron content which is inorganic iron with low bioavailability (1–12%), without counting the possible iron leaching during cooking [86,88]. Iron bioavailability is moreover hampered by dietary fiber, and phytate but increased by vitamin C. Reported intakes, shown in Figure 2, were generally comparable or even higher in children following a vegetarian, a vegan, or a macrobiotic diet than in those omnivorous [35,36,37,39,40,41,42,44,46,47,48,49,74,95].

However, none of these studies considered iron bioavailability. As a consequence, when assessed, the iron store was shown reduced [35,36,38,39,42,44,48,74]. This is even though it is acknowledged that iron absorption is increased when iron status is deficient which leads to hepcidin suppression [86]. Few studies reported lower hemoglobin concentration [36,38]. Rare cases of iron deficiency anemia due to a vegetarian diet have been reported [41,44,96]. The risk of iron deficiency is increased in the event of an unfavorable socio-economic situation [86].

### 4.2. Zinc

**Figure 3:**
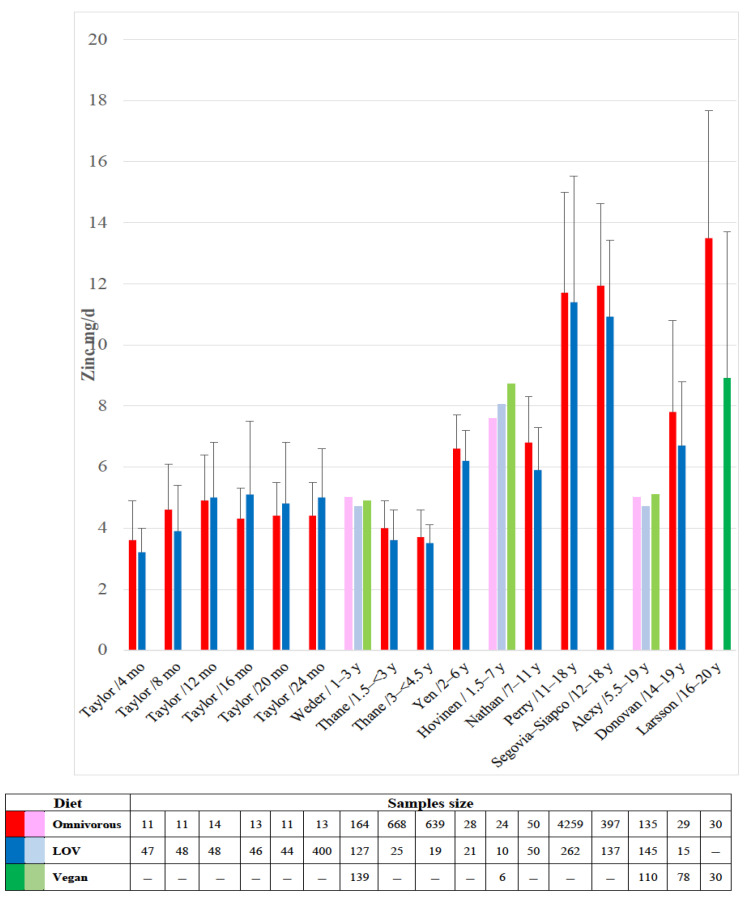
Bar chart to present the reported zinc intake in lacto-ovo-vegetarian (LOV)and vegan children compared to omnivorous counterparts [36,37,39,40,41,42,46,47,48,49,95]. The dark-colored bars display the mean with SD, when available, whereas the light-colored bars display the median value.

Zinc provides the prosthetic group of several enzymes to assist in the most major metabolic pathways and is involved in the receptor proteins for vitamins A and D, as well as for thyroid and steroid hormones [87,97]. Worldwide, it has been estimated that 17% of the population may have inadequate zinc intake with a lower percentage in high-income countries than in low-income regions [98]. In this survey, on average 34.8 ± 20% of zinc intake was from ASF. The highest zinc content is found in oysters, shellfish, and red meat, whereas FPO providing the most zinc are whole grains, fortified cereals, pulses, nuts, and seeds [76,97]. However, as for iron, the rich content of phytate, oxalate, or fiber in FPO may interfere with zinc absorption [87,97]. Total dietary phytate and the phytate/zinc molar ratio were shown positively correlated with the risk of inadequate zinc intake (r = 0.62 and 0.92, respectively; *p* < 0.01) [98]. These considerations were not considered in the studies reported in Figure 3.

The reported intakes of vegetarian or vegan children were similar to that of omnivorous counterparts in most of these studies [37,39,40,42,47,49,95], with similar plasma zinc concentrations [39,47]. However, in three studies, the intake was lower in vegetarian children than in omnivores [36,41,46]. None of these studies reported clinical features of zinc deficiency but some case reports in young vegan children fed with plant milk [96]. The clinical setting of zinc deficiency may range from poor appetite and anorexia, decreased growth velocity, increased susceptibility to infection, diarrhea, depressed mood, and skin rashes at orifices [90].

### 4.3. Iodine

Iodine is a mandatory structural and functional element of thyroid hormones and, subsequently, has an important role in the growth, development of neurological, and cognitive functions [99,100]. The iodine concentration, as iodide, in water and foods is highly variable.

The main iodine sources are marine products, eggs, milk, and iodized salt. In meats as well as in FPO the iodine content depends on the richness of the soil of the food production region [90,94]. Adults who consume diets excluding iodine-rich foods have been shown to have an increased risk of iodine deficiency [100,101]. Studies assessing iodine intake in vegetarian children are scarce. A German study found that LOV children had lower intake than omnivores [49]. This was not found in a few Finnish young vegan children who also had similar iodine urine concentration to that of omnivores [47].

### 4.4. Other Microminerals

Although variably provided by plants, these micromineral intakes have very rarely been evaluated in vegetarian children. Apart from ASF, they may be mainly provided by grains legumes and seeds depending on the soil content [32,102]. Copper intake has been found to be similar or higher in vegetarian children in two studies [37,95]. Canadian LOV adolescents had higher manganese intake than their omnivorous counterparts [37]. Selenium intake in Swedish vegan adolescents were lower than that of omnivorous adolescents [41]. Apart from these few studies, no other study was found in particular regarding the intake of chromium or molybdenum.

## 5. Discussion and Recommendations

This review emphasizes that the more children are subjected to a diet restricted in ASF (vegan or macrobiotic), the more they are at risk of developing a deficiency in certain micronutrients. All micronutrients, especially vitamin B12 cannot be obtained in an adequate amount from non-ASF. Moreover, some such as carotenoids which can be found as precursors in FPO have a poor conversion to their active form (retinol) and others (e.g., iron and zinc) display low bioavailability. However available data from the studies carried out so far do not formally allow us to find a clear relationship between the diet, which can have diverse patterns, and the intake or the status of micronutrients. There are several reasons for this, some of which represent the limitations of this review, which should be acknowledged.

### 5.1. Limitations

Many of the reported studies are relatively old, with 53% of them conducted more than 25 years ago [35,36,37,38,39,40,41,77,95], and only a third less than 10 years ago [45,46,47,48,49,74,78]. All are observational studies, frequently enrolling a quite small number of children based on the voluntary participation of the parents. Parents who are likely to be concerned about their own health and that of their child and who are better able to practice a balanced diet and give supplements. The use of such supplements is rarely specified in the calculation of intake. Little information is provided about the duration of a vegetarian or a vegan diet in the various population groups, whereas there is inconsistency in the definition of vegetarianism, leading to a great heterogeneity in the vegetarian spectrum and a great dispersion in the results as shown in the figures. The heterogeneity of the results may also be linked to the use of different modes of assessment of intakes, which may use a food frequency questionnaire or a dietary record on one or three days. Moreover, most of the studies have been conducted in industrialized and high-income countries and neither socioeconomic status nor the environment was considered. Last but not least, very few studies have set the evidence of micronutrient shortfall by performing the appropriate tests in blood or urine samples and thus assessing the child’s micronutrient status.

The nutritional adequacy of a vegetarian diet must be judged individually, given that a vegetarian diet can be composed in many different ways. On the other hand, in addition to the concern about micronutrients, other disadvantages, and risks must be analyzed. Of additional concern are energy, protein, *n*-3 long chain-polyunsaturated fatty acids, and calcium intake as well as protein quality and bone health.

### 5.2. Recommendations

From an ethical point of view, the food choice of parents must be respected, whatever the reason. Health professionals must inform parents of the possible risks of an ill-conceived vegetarian diet, but also of the possible benefits in terms of the health of a well-balanced diet. The diet and physical status of pregnant and nursing women and of children or adolescents following a restrictive diet must be carefully monitored and the help of a trained nutritionist may be needed. The main challenge for practitioners is to assess the risk of nutritional deficiency and to determine the need for laboratory tests.

In descending order, the most inappropriate ASF-restrictive diets are the macrobiotic, the vegan, the pescatarian, the lacto-vegetarian, the ovo-vegetarian, the LOV, and the flexitarian diets. A well-balanced LOV diet may be a healthy choice if the potential nutrient deficits are recognized and acted upon. Several dietary guidelines on vegetarian and vegan diets have been developed in this regard [103,104,105,106,107].

Vegan mothers, infants, and children may require supplementation with vitamin B12, iron, and zinc [76,107,108,109,110,111]. Exclusive breastfeeding is recommended for the 1st 4–6 months provided that the mother is supplemented with vitamin B12. Otherwise, an infant formula must be given, followed after the introduction of complementary food by a follow-on-formula, then a young child formula, which should be used as long as possible up to three years of age [112,113]. In cases of refusal of cow milk-based formulae, an alternative is a soya or hydrolyzed rice infant formula [114]. However, industrial or home-made preparations incorrectly named “plant-based milk”, i.e., non-dairy beverages made from a water-based plant extract such as rice, soya, almond, coconut, grains, or other extracts must be strictly avoided because they are not suitable for feeding young children [96].

Complementary feeding should be carefully planned to ensure all critical nutrients are provided in agreement with current nutritional recommendations, and this is quite challenging [26,76,107,108,110,112,115,116,117,118,119]. It should include a wide variety of foods (grains, legumes, pulses, vegetables, fruit, nuts, and seeds). The use of iron- or bio-fortified food should be encouraged [76,86,110]. Apart from taking supplements, it is much more difficult or even impossible for vegans to ensure an adequate intake of vitamin B12 and iron. On the other hand, vitamin D supplementation should be recommended in all children, especially infants and adolescents, whatever their diet [51,53,54].

Parents and adolescents knowledge of nutrition, with which habits and beliefs interfere, determine their food choices according to the availability of certain foods and the subsequent nutrition adequacy of any one particular plant-based diet. This highlights the importance for health professionals to inform and advise families who follow a restrictive diet. Pregnant women, newborns, children, and adolescents with a vegetarian dietary pattern need medical supervision. The underlying problem is the frequent ignorance of the ins and outs of such diets and the solutions to be provided. This is the result of a lack of nutritional education and training in medical schools and postgraduate courses [118,119,120,121,122].

## 6. Conclusions

Since children’s diets are largely driven by their parents, the prevalence of vegetarian diets during childhood is certainly on the rise parallel in industrialized countries. Maintenance of a vegetarian diet can be challenging. The risk of micronutrient deficiency in vegetarian children is relatively difficult to assert based on the current limitations of evidence due to the lack of well-designed studies. This means the need for more adequately powered trials to better identify any problems, which are not easy to conduct.

However, the risk of vitamin B12 deficiency must be considered in newborns from vegan or macrobiotic mothers and children with severe restrictions of ASF. Iron deficiency needs to be assessed individually in vegan children, as well as that of iodine and zinc. For this, some appropriate tests can be carried out, bearing in mind that the more restrictive the diet in ASF, the greater the risk. A LOV diet exposure is a low risk as long as it uses a very varied diet with a sufficient intake of dairy products and especially specific formula in infants and young children. Most of the deficiencies may be preventable through nutrition guidance and the consumption of a well-planned diet containing diverse foods, as well as food fortification and supplementation, where needed. On the other hand, it would be better to avoid vegan and macrobiotic diets during pregnancy and childhood. However, when it comes to micronutrients, special attention should be paid to vitamin B12, iron, zinc, iodine, and vitamin D.

Parents and teenagers must be informed of the serious consequences of failing to follow the advice and prescriptions regarding supplementation of the diet and the need for medical and dietic regular supervision.

## Data Availability

Not applicable.

## Abbreviations

ASF	animal food sources
FPO	foods of plant origin
LOV	lacto-ovo-vegetarian
